# Pediatric Transplant Surgeons' Perspectives on Palliative Care for Children With Chronic Kidney Disease: A National Cross‐Sectional Survey

**DOI:** 10.1111/petr.70037

**Published:** 2025-01-28

**Authors:** Taylor R. House, Aaron Wightman, Jodi Smith, Andre Dick, Miranda C. Bradford, Abby R. Rosenberg

**Affiliations:** ^1^ Department of Pediatrics University of Wisconsin Madison, School of Medicine and Public Health Madison Wisconsin USA; ^2^ Department of Pediatrics University of Washington, Seattle Children's Hospital Seattle Washington USA; ^3^ Department of Surgery University of Washington, Seattle Children's Hospital Seattle Washington USA; ^4^ Biostatistics Epidemiology and Analytics in Research Core, Seattle Children's Hospital Seattle Washington USA; ^5^ Department of Psychosocial Oncology and Palliative Care Dana‐Farber Cancer Institute Boston Massachusetts USA; ^6^ Department of Pediatrics, Pediatric Advanced Care Team Boston Children's Hospital Boston Massachusetts USA; ^7^ Department of Pediatrics, Division of Hematology‐Oncology Harvard Medical School Boston Massachusetts USA

**Keywords:** palliative care, pediatric chronic kidney disease, pediatric kidney transplant

## Abstract

**Background:**

Some adult transplant surgeons consider transplant to be contraindicated in patients receiving palliative care (PC). Little is known about pediatric transplant surgeons' attitudes toward PC. We sought to ascertain pediatric kidney transplant surgeons' perspectives regarding the routine integration of PC for children with chronic kidney disease.

**Method:**

We administered a cross‐sectional web‐based survey to members of the American Society of Transplant Surgeons listserv in summer 2021. We adapted the survey from the previously validated Provider Survey about Palliative Care for Children with Heart Disease and pretested it with representative kidney transplant surgeons, nephrologists, and PC physicians; queries related to PC included institutional and personal experience, knowledge, and education. Data were summarized descriptively.

**Results:**

There were 21 participants. Over half of the respondents were white (57%) males (62%), practicing in urban, academic centers (94%). Although 67% of the participants practiced in an institution with a subspecialty PC team, 24% were unsure if such a team existed in their institution. A minority (19%) perceived PC consultation and kidney transplant to be mutually exclusive. Most surgeons (86%) believed that PC should *not* be restricted to when a child is dying, and 59% reported that PC consultation should happen at diagnosis for life‐threatening conditions. However, surgeons indicated that PC consultation is rarely utilized for pediatric kidney transplant recipients. Transplant surgeons expressed a desire for additional PC‐focused training and willingness to engage in additional education.

**Conclusions:**

Although a minority of pediatric transplant surgeons perceived PC to be contraindicated for kidney transplant, most indicated openness to PC engagement for their patients.

**Abbreviations**
CKD: chronic kidney disease
PC: palliative care

## 1 Introduction

Children with chronic kidney disease (CKD) and their families encounter significant impediments to flourishing related to their illness [1]. Compared to healthy, age‐matched peers, the death rates among children with advanced CKD and those who have received kidney transplants are about 30 and 13 times higher, respectively [2, 3]. Physical symptoms including pain, gastrointestinal distress, fatigue, sleep disturbances, pruritis, thirst, and edema are highly prevalent [1, 4]. Poor mental health is also pervasive and affects up to half of children with CKD [5, 6]. With frequent fluctuations in health and the challenges inherent to managing a chronic illness, children with CKD and their families also require high‐quality serious illness communication and may encounter difficulty in engaging in care plan development with their clinicians [7, 8, 9, 10]. These burdens are not limited to the impacted child and extend to siblings and caregivers during and following transplant [1, 11, 12, 13]. Palliative care (PC), holistic care delivery focused on alleviating symptoms and stress accompanying a serious illness and improving quality of life for the child and family, offers the chance to alleviate these myriad burdens [14, 15]. Importantly, this care is indicated from the time of serious illness diagnosis and may occur concurrently with disease‐directed treatments.

Kidney transplant is recognized as the ideal treatment for children with advanced CKD. As such, pediatric transplant surgeons serve a vital role in care often over years of a child's life; spanning pretransplant evaluation, admission for the transplant procedure, and longitudinal follow‐up in posttransplant management. This influential role of pediatric transplant surgeons at high‐stakes points in care makes their perspectives on PC integration particularly important [1]. Some adult transplant surgeons view specialty PC engagement as a contraindication for organ transplant candidates and conflate PC with hospice or end‐of‐life care. Because of this perception, some transplant candidates are denied the opportunity to receive PC [16, 17, 18, 19]. Little is known about pediatric transplant surgeons' attitudes toward PC in pediatric nephrology. We sought to ascertain pediatric transplant surgeons' perspectives regarding routine integration of PC for children with CKD.

## 2 Materials and Method

We performed an observational, anonymous, cross‐sectional web‐based survey study of pediatric transplant surgeons in the United States. We electronically distributed the survey to pediatric transplant surgeons associated with the American Society of Transplant Surgeons (ASTS) listserv from August to September 2021. We obtained surgeons' consent to participate in the first question of the survey. To confirm their eligibility to participate, we required participants to respond to three mandatory questions identifying themselves as transplant surgeons who perform pediatric kidney transplants and practice in the United States. The survey closed automatically if these inclusion criteria were not met or consent was not provided. We sent an initial email introducing the study and survey. We then sent reminder emails to participate two and 3 weeks following the initial email. The study was determined to be exempt from review by the Seattle Children's Hospital Institutional Review Board.

We adapted the survey from the Provider Survey about PC for Children with Heart Disease, a previously validated survey tool used to investigate pediatric cardiology physicians' and cardiothoracic surgeons' attitudes about PC [20, 21]. The survey primarily included close‐ended questions with multiple‐choice and 5‐point Likert response options. As we have previously described in adapting and administering this survey to pediatric nephrology fellows, we modified the survey for pediatric transplant surgeons, first performing a review of pertinent pediatric PC and kidney transplant literature [22]. After adaptation based on the literature review, we convened a panel of three pediatric PC physicians, three pediatric nephrologists, one of whom is a kidney transplant director, and two pediatric transplant surgeons to review the survey content and design. We beta‐tested the survey with three representative pediatric transplant surgeons, performed debriefings, made modifications, and established the final survey [23]. Domains included demographic and practice characteristics, institutional and personal PC experience, and PC knowledge and training. We also specifically queried (a) surgeons' knowledge of PC principles (5‐point Likert‐scale with options ranging from “no” = 1 to “extensive” = 5 knowledge and a multiple‐choice question investigating how surgeons acquired such knowledge); (b) perspectives on the indications and role of subspecialty PC consultations and resources (5‐point Likert‐scale with options ranging from “strongly disagree” to “strongly agree”); (c) aspects of local PC resources including timing and criteria for automatic PC consultation (multiple‐choice and timing options including “too early,” “at the appropriate time,” “too late,” and “contact too limited to assess”); (d) subspecialty PC team involvement with kidney transplant recipients (multiple‐choice and 5‐point Likert‐scale with options ranging from “never” to “always” in questions of the extent to which the subspecialty PC team is involved with potential or prior pediatric kidney transplant recipients); (e) surgeons' opinions regarding which clinicians should be responsible for leading goals of care conversations for pediatric nephrology patients across healthcare settings ranging from outpatient to intensive care (multiple‐choice); and (f) domains of PC delivery in which surgeons could benefit from additional training, their preferred formats for future educational opportunities, and ideal timing for PC training (multiple‐choice, 5‐point Likert‐scale with options ranging from “very unlikely” to “very likely” in questions of how likely surgeons are to participate in proposed PC learning opportunities). We also obtained demographic information including years since medical school graduation, gender, race, ethnicity, hospital size, practice geographic location, the types of solid organs participants routinely transplant, primary patient population (pediatric, adult, or combined), and kidney transplant volumes at their institution.

We used REDCap electronic data capture tools hosted at Seattle Children's Hospital to collect and manage data [24, 25]. We summarized responses descriptively using counts, percentages, and graphical representations. Analyses were performed using Stata version 16 (StataCorp, College Station, TX). For questions regarding surgeon perspectives on indications and benefits of PC consultation and resources, responses were dichotomized as “agree” (“agree” or “strongly agree” response selections) versus “disagree” (“disagree” or “strongly disagree” response selections). For questions focused on participation in future PC training activities, responses were similarly dichotomized as “likely” (“likely” or “very likely” response selections) versus “unlikely” (“unlikely” or “very unlikely” response selections).

## 3 Results

There were 21 responding transplant surgeons. Participants were not required to answer every question, so some items had response totals less than 21. Demographic and practice characteristics of the respondents are shown (Table 1). Participants were 57% white, 62% male, and most (94%) practice in academic, urban centers. Geographic practice location within the United States varied, with 17% practicing in the Northeast, 28% in the Midwest, 33% in the South, and 22% in the West. Half of surgeons were affiliated with hospitals with > 200 inpatient pediatric beds, and 61% were in practices where ≤ 20 kidney transplants are performed annually. Although 67% of transplant surgeons practice in an institution with a pediatric PC service, 10% did not, and 24% were unsure if such a team exists at their hospital. Only 4 surgeons' (19%) surgical practice was composed of primarily pediatric patients. All respondents performed kidney transplants. Many (62%) also performed liver transplants, and some (24%) performed intestinal transplants. Half completed pediatric specific training following surgical residency.

**TABLE 1:** Participant characteristics.

	Total (*N* = 21)*
Sex, *n* (%)	
Female	8 (38)
Male	13 (62)
Race, *n* (%)	
White	12 (57)
Black	5 (24)
Asian	2 (10)
Other race not American Indian, Pacific Islander, Native Hawaiian, or Alaskan native	2 (10)
Ethnicity, *n* (%)	
Hispanic or Latino	5 (24)
Practice location, *n* (%)	
Northeast	3 (17)
South	6 (33)
Midwest	5 (28)
West	4 (22)
Pediatric kidney transplants performed annually hospital‐wide, *n* (%)	
None	2 (11)
< 10 transplants	4 (22)
10–20 transplants	5 (28)
21–30 transplants	4 (22)
> 30 transplants	3 (17)
Access to palliative care team, *n* (%)	
Yes	14 (67)
No	2 (10)
Unsure	5 (24)
Completed ≥ 6 months of pediatric specific training, *n* (%)	
Yes	10 (50)
Primary patient population, *n* (%)	
Pediatric	4 (19)
Adult	7 (33)
Both	10 (48)

* Participants were not required to answer every question; responses may not total 21.

Surgeons reported a low mean knowledge of PC principles of 2.5 ± 0.8. For surgeons who described having knowledge of PC principles, the most common way this knowledge was acquired was through experience with PC consultation for their patients (81%), and 86% found prior PC consults to be helpful. When asked about their perceptions of PC involvement, a minority of surgeons (19%) perceived PC consultation and kidney transplant to be mutually exclusive, and 67% of surgeons disagreed with this sentiment.

**FIGURE 1:**
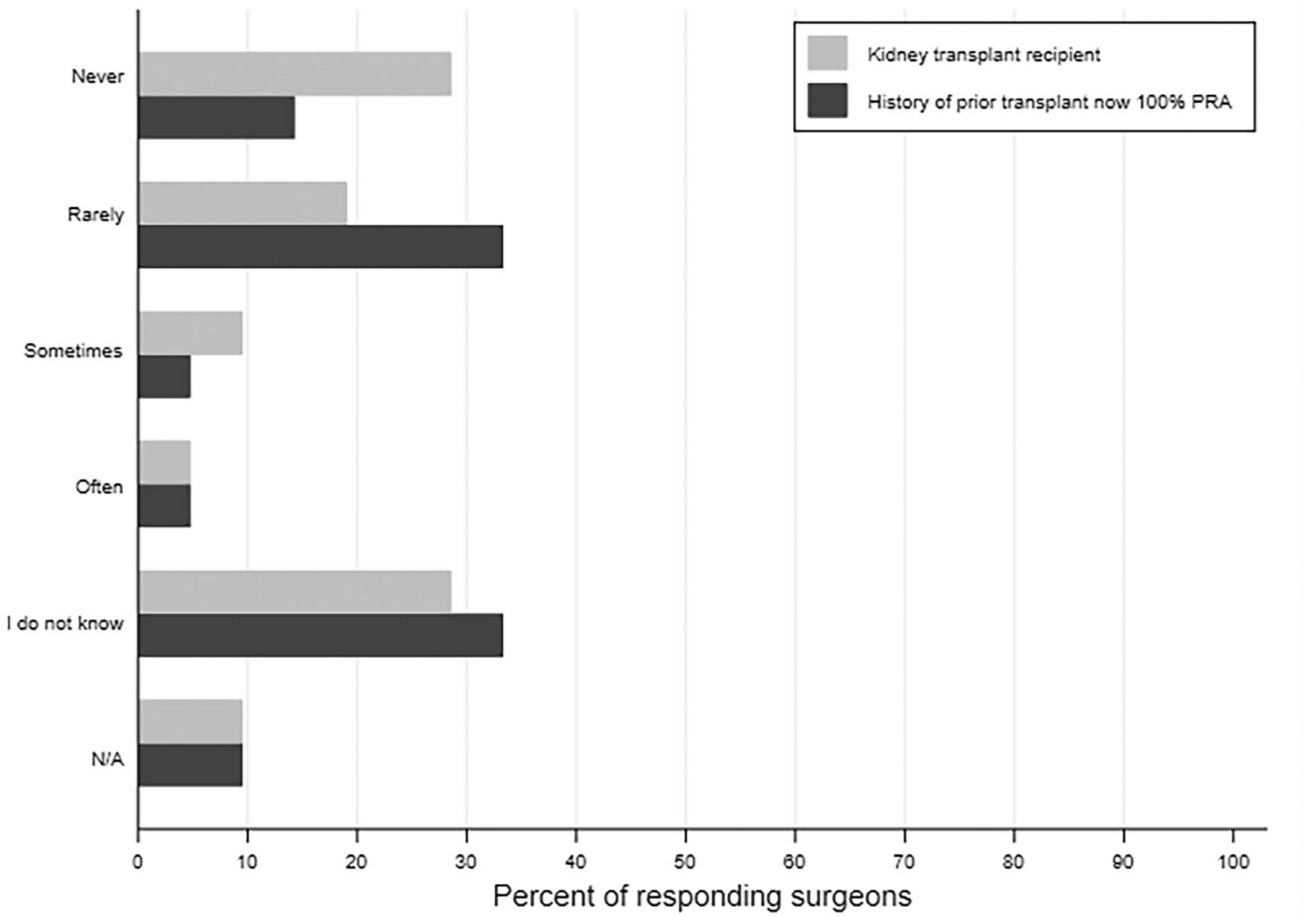
PC involvement for pediatric kidney transplant recipients. Surgeons (*n* = 21) report infrequent PC team involvement among pediatric kidney transplant recipients, including those who are highly sensitized. A portion of transplant surgeons was also unsure of the frequency of PC consults in this population. PC, palliative care.

**FIGURE 2:**
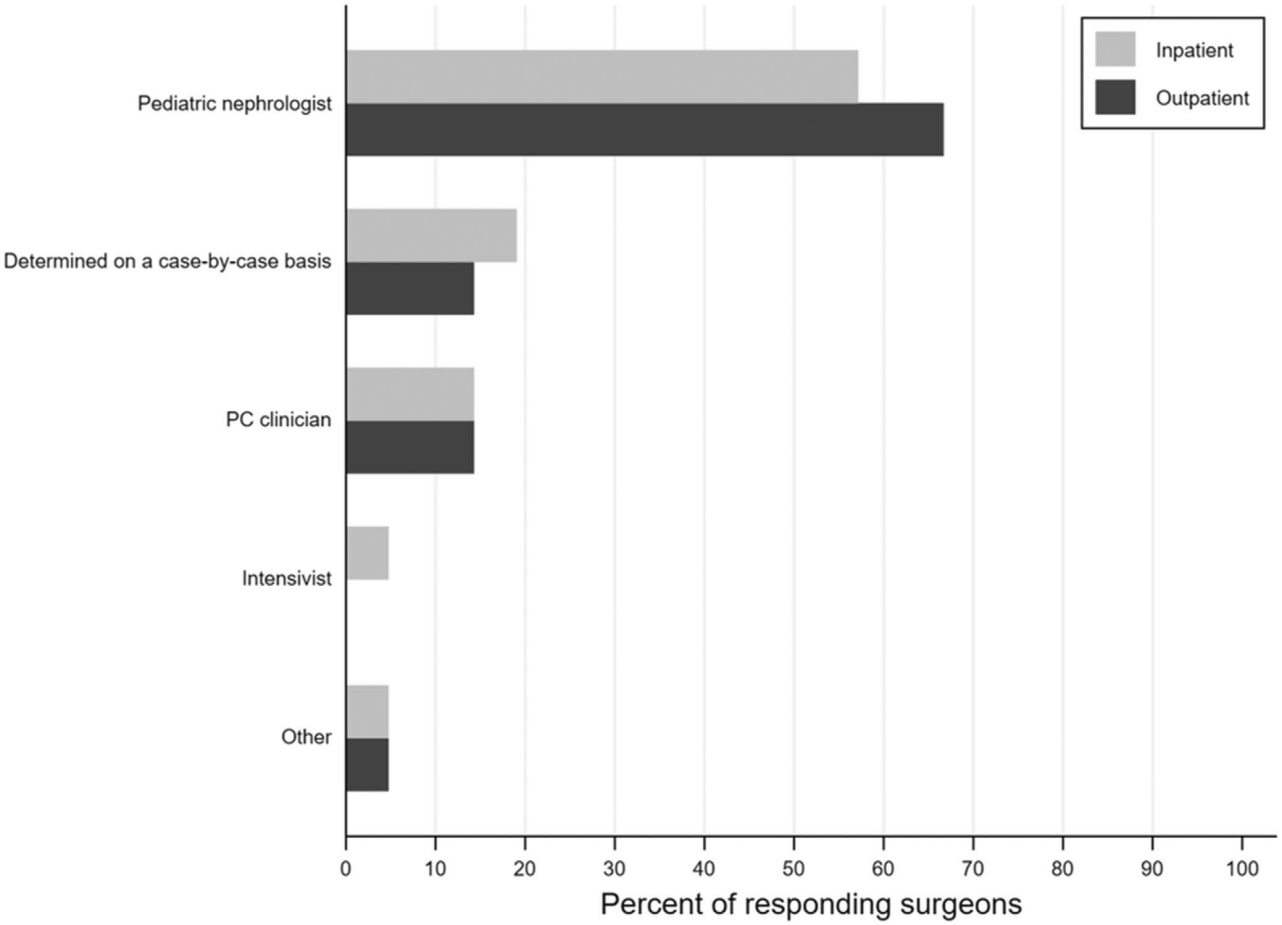
Transplant surgeons' perspectives on responsibility for serious illness communication among pediatric kidney transplant recipients. Among responding transplant surgeons (*n* = 21), most indicate that pediatric nephrologists should have the primary responsibility for discussing goals of care with pediatric kidney transplant recipients and their families in inpatient and outpatient healthcare settings.

Almost half of surgeons (43%) felt that PC consults occur too late at their institution. Most transplant surgeons (86%) believed that PC should *not* be restricted to when a child is dying, and 59% responded that PC consultations should happen at the time of diagnosis for life‐threatening conditions where a cure is possible and may fail. Yet, 48% of surgeons reported that the PC service is never or rarely consulted for kidney transplant recipients or prior kidney transplant recipients now highly sensitized (Figure 1). About a third of surgeons were unsure of the frequency with which the PC team is involved in either of these scenarios (29% and 33%, respectively). Surgeons most commonly identified pediatric nephrologists as primarily responsible for serious illness communication, including goals of care for pediatric kidney transplant recipients across healthcare settings. Sixty‐seven percent believed that nephrologists should fulfill this need in an outpatient setting and 57% indicated the same in an inpatient setting (Figure 2).

Transplant surgeons expressed an interest in additional PC training. Nearly half (44%) of respondents indicated they would benefit from additional training in serious illness communication pertaining to prognosis and in the management of psychological distress of children. Most surgeons thought this type of training should happen during surgical residency (78%), and 61% were also receptive to training as attending surgeons. The most popular formats for future training included grand rounds sessions focused on PC in pediatric nephrology or web‐based modules for continuing medical education, with 67% and 50% reporting that they were likely to participate in this type of training, respectively.

## 4 Discussion

In this national sample of pediatric transplant surgeons, we found that surgeons may be unfamiliar with the availability of or need for PC resources among children requiring kidney transplants. Although a minority of transplant surgeons view receipt of PC as a contraindication to kidney transplant, surgeons generally demonstrated an openness to and interest in the benefits of PC for pediatric kidney transplant recipients, particularly when led by pediatric nephrologists.

Transplant surgeons serve an instrumental role in the lives of patients and families before, during, and following kidney transplant, a period characterized by high PC needs. Prior to kidney transplant, surgeons are engaged in transplant evaluation and the identification of families who are prepared to proceed with the onerous transplant journey. The transplant procedure may be an intense time of high worry, uncertainty, powerlessness, and hope. Balancing these conflicting emotions, a significant operation, and the possibility of a living related donor as an added complexity, transplant is a crucial point with deep engagement between the family and the transplant team. Following the operation, transplant surgeons continue to see transplant recipients in the clinic and support their navigation of the new opportunities and challenges that transplant may present. As such, their support is critical to successful PC integration for children with advanced CKD.

Our findings demonstrate that transplant surgeons may have limited awareness of the PC resources available to support pediatric kidney transplant recipients and their need for such resources. A proportion of surgeons were unsure if their hospital had a pediatric PC team and were unaware of the frequency with which PC consults are pursued among pediatric kidney transplant recipients. Over half of respondents indicated that PC consultations should occur at the time of diagnosis for life‐threatening conditions where cure is possible and may fail; but about half also reported that the PC service is never or rarely consulted for pediatric kidney transplant candidates or prior recipients who are now highly sensitized. This raises two important considerations. First, transplant surgeons may not perceive advanced CKD and the need for kidney transplant to be a life‐threatening condition and therefore not in need of PC resources. In adult medicine, the benefit of integrated PC is increasingly being realized for illnesses outside of cancer and timing outside of the end‐of‐life period. However, PC continues to be underutilized in adult cases of organ transplantation [16, 17, 18, 19]. Our findings mirror those in adult kidney transplant medicine, where studies of PC for kidney transplant recipients have historically focused on those whose grafts are already failing or have failed [19]. Prior studies have shown that surgeons may perceive the PC team to be a “death service” or the philosophy of PC to be “giving up” while the emphasis of surgery is to “never say die” [17, 18]. Although kidney disease is unable to be cured, surgeons carry a strong emphasis on cure and the “lifesaving ethos” of surgery, in turn perpetuating the misconception that PC and transplant cannot be pursued concurrently, as demonstrated by a minority of our respondents [26]. This does a disservice to pediatric kidney transplant recipients as they encounter significant burdens of their disease [1]. Further, adolescent kidney transplant recipients in particular may benefit from earlier PC support to preemptively combat their disproportionate high rates of graft failure [3, 27].

Alternatively, this finding of infrequent PC consultation among pediatric kidney transplant recipients, even though surgeons believe PC consultations should occur at diagnosis for life‐threatening conditions, may suggest that surgeons are open to engagement of PC for children with advanced CKD but that they rely upon pediatric colleagues, and particularly pediatric nephrologists, to initiate PC supports. This is reinforced by the finding that surgeons most frequently identified pediatric nephrologists as the clinicians primarily responsible for providing serious illness communication to pediatric kidney transplant recipients. Several surgeons also described PC consults as occurring “too late” at their institution. Taken together, our findings suggest that transplant surgeons may be supportive of the initiation of PC for pediatric kidney transplant recipients when led by pediatric nephrologists but are uncertain as to the optimal timing or circumstance in which PC is indicated. Murakami et al. [19] recently found delayed PC consultation among adults with failing kidney transplants due to transplant clinicians being unsure of the ideal time or circumstance for PC integration. We have previously proposed PC integration into the care of all children with CKD at predefined inflection points, including surrounding kidney transplant, to alleviate delayed PC delivery and clinician concerns about the perception of PC consultation [1, 28]. Further, utilizing pediatric nephrologists with an expanded PC skillset can help to facilitate earlier, standardized PC delivery to best support patient and family flourishing [1, 28]. Surgeons' enthusiasm for PC engagement will need to be supported by pediatric nephrologists who spearhead identification of PC needs and initiation of PC resources.

These findings may also be important for other pediatric solid organ transplant populations. About two‐thirds (62%) of respondents performed liver transplants and one‐quarter (24%) performed intestinal transplants. Although our study did not investigate surgeons' perspectives on PC for these specific groups, like children with kidney transplants, they similarly experience life‐threatening conditions. Support for PC consultations at diagnosis for life‐threatening conditions among surgeons in this study coincides with an increasing recognition of the need for PC engagement among children who require other types of solid organ transplants [16, 29, 30, 31]. Our findings may foretell surgeons' support of PC engagement among children requiring liver or intestinal transplants and that PC incorporation is most likely to be successful when initiated by pediatric subspecialists like hepatologists and nephrologists [32, 33, 34].

Nearly half of transplant surgeons were interested in receiving additional training in communication and psychosocial distress aspects of PC delivery. Although residency is the preferred time for this type of training, there was an openness to PC education as an attending, as well. This mirrors findings among surgeons who care for children with advanced heart disease [21]. This need could be addressed through the adaptation of existing communication curriculum such as NephroTalk. NephroTalk is an educational program developed in response to low levels of confidence to engage in serious illness communication procedures among adult nephrology fellows and designed to enhance the PC skills of nephrologists. Future work should focus on the development of such potential tools to increase awareness of PC resources and bolster the PC skills of transplant surgeons.

Our study has some important limitations. First, our sample size is small. The ASTS listserv is comprised of 775 transplant professionals, including solid organ transplant surgeons, physicians, and researchers, about 360 of whom are estimated to perform pediatric kidney transplants [35]. Although we were unable to calculate a response rate, our sample likely represents a small portion of this population. The limited number of respondents may indicate that transplant surgeons are unaware of the PC needs of children with CKD or do not think it is a significant issue. This reinforces the importance of our study's findings. Because of the small sample size, generalizability is limited. Transplant surgeons who responded to the survey may differ from nonrespondents in their experience with or interest in PC. Most of our participants were white males who practice at urban academic centers. Perspectives of diverse clinicians who practice in rural or community settings may be lacking, which is of particular interest as these centers may have lower access to specialty PC services [36, 37].

Our study provides important and previously unreported insights into the perspectives of transplant surgeons attitudes toward PC for children with advanced CKD. Although a small subset of transplant surgeons continues to view PC and kidney transplant as mutually exclusive, many are receptive to PC for children with advanced CKD and are interested in expanding their PC skill set. Due to a lack of familiarity with PC resources for this population among transplant surgeons, pediatric nephrologists may need to initiate PC services and serve as a liaison between transplant surgeons, PC clinicians, and children with advanced CKD and their families. Future research should include intervention development to standardize PC integration for children with advanced CKD into kidney transplant care and break down silos of expertise between pediatric nephrologists, PC physicians, and transplant surgeons.

## Conflicts of Interest

The authors declare no conflicts of interest.

## Data Availability

The data that support the findings of this study are available from the corresponding author upon reasonable request.
